# The cation-π interaction in cysteine-rich domain of Smoothened is critical for its cholesterylation and function: The cation-π interaction is required for SMO cholesterylation

**DOI:** 10.3724/abbs.2022090

**Published:** 2022-07-27

**Authors:** Zekai Kong, Min Xu, Yanqing Zhang, Wenda Huang, Xiaolu Zhao, Jie Luo, Bao-Liang Song

**Affiliations:** Hubei Key Laboratory of Cell Homeostasis College of Life Sciences TaiKang Center for Life and Medical Sciences TaiKang Medical School Wuhan University Wuhan 430071 China

**Keywords:** Hedgehog, Smoothened, cholesterylation, cysteine-rich domain, cation-π interaction

## Abstract

The Hedgehog (Hh) signaling pathway is critical for embryonic development and tissue renewal. The G protein-coupled receptor (GPCR)-like protein Smoothened (SMO) is the central signal transducer in the Hh pathway. Cholesterol binds and then covalently links to the D95 residue of cysteine-rich domain (CRD) of human SMO. The cholesterylation of CRD is critical for SMO activation. SMO cholesterylation is a Ca ^2+^-boosted autoreaction that requires the formation of an ester bond between the side chains of D95 and Y130 as an intermediate. It is unknown whether other residues of SMO are involved in the esterification between D95 and cholesterol. In this study, we find that the SMO-CRD(27–192) can undergo cholesterylation. In addition to D95 and Y130, the residues critical for cholesterol modification include Y85, T88, T90, W109, W119, K133, E160 and F166. T88, W109, W119 and F166 also seem to be involved in protein folding. Notably, we find that Y85 and K133 form a cation-π interaction whose disruption abolishes cholesterylation and ciliary localization of SMO. This study highlights the mechanism and function of cholesterol modification of SMO.

## Introduction

The Hedgehog (Hh) signaling pathway controls embryonic development and tissue homeostasis [ 1– 3] . Aberrant activation of Hh signaling causes multiple types of cancers including basal cell carcinoma and medulloblastoma [4]. The Hh signaling pathway has three major players: the ligand Hh, the receptor Patched-1 (PTCH1), and the G protein-coupled receptor (GPCR)-like protein Smoothened (SMO) [ 5– 7] . Hh directly binds to PTCH1 and derepresses SMO [8]. SMO then translocates to primary cilium and finally activates the transcription factors GLIs, eventually stimulating the expression of many target genes [ 7, 9– 11] .

Cholesterol plays an essential role in the regulation of SMO. The human SMO protein is composed of a signal peptide (1–26 aa), an extracellular/luminal cysteine-rich domain (CRD) (27–192 aa) and a seven-transmembrane (7TM) domain. The R451 residue in TM6 forms a cation-π interaction with the W535 residue in TM7, thereby locking SMO in an inactive state. This cation-π interaction, however, is broken upon SMO activation. The W535L mutation constitutively activates SMO and causes sporadic basal cell carcinoma [12]. Cholesterol can trigger SMO activation by binding to the CRD or 7TM domain [ 13– 20] .

We previously showed that SMO is covalently modified by cholesterol through an ester linkage between the 3-beta hydroxyl group of cholesterol and the side chain of D95 in human SMO (D99 in mouse SMO) [ 7, 21] . We later demonstrated that cholesterol binding to the CRD is not sufficient to trigger SMO activation because the SMO(D95E) mutant, which can bind to but fail to be covalently modified by cholesterol, is inactive and associated with severe developmental defects [22]. Mechanistically, the cholesterylation of SMO-CRD is a Ca ^2+^-boosted autoreaction that requires an intramolecular ester bond formed between the side chains of D95 and Y130, which serves as a high-energy intermediate [22].

To understand the biochemical mechanism of SMO cholesterylation, we systematically mutated 66 residues in CRD that contain the functional groups in their side chains. We found that the cation-π interaction between the Y85 and K133 residues is essential for SMO cholesterylation and function.

## Materials and Methods

### Reagents

The mouse mAb anti-SMO clone E5 (sc-166685) was from Santa Cruz (Santa Cruz, USA). The mouse mAb anti-FLAG (clone M2, F3165) was from Sigma (St Louis, USA). The rabbit pAb anti-ARL13B (17711-1-AP) was from proteintech (Rosemont, USA). The Alexa Fluor 488 goat anti-mouse secondary antibody (A11001) and Alexa Fluor 555 donkey anti-rabbit secondary antibody (A31572) were from Thermo Scientific (Waltham, USA). The donkey anti-mouse HRP-conjugated secondary antibody (715-035-150) was from Jackson ImmunoResearch (West Grove, USA). CuSO _4_ (C8027), Vc (A7631) and TBTA (678937) were from Sigma. Cholesterol probe (CP) and biotin alkyne were synthesized by Shanghai Bio Bond Pharmaceutical Co., Ltd (Shanghai, China) [21].

### Cell culture

HEK293T and NIH3T3 cells (ATCC, Manassas, USA) were cultured in DMEM containing 10% FBS (ExCell, Shanghai, China), 100 units/mL penicillin, and 100 μg/mL streptomycin sulfate at 37°C in 5% CO _2_.

### Plasmids

The open reading frame of human SMO cDNA was amplified from HEK293T cell, and pCMV-SMO-WT was constructed by inserting cDNA into pcDNA3. pCMV-SMO(1–259)-FLAG, pCMV-SMO(1–233)-FLAG and pCMV-SMO(1–192)-FLAG were generated by PCR, followed by insertion of a 3×Flag sequence after the truncation of SMO. All mutants of SMO plasmids were constructed by the quick-change method [ 23, 24] .

### Click chemistry

The experiments were performed as previously described [25]. Samples were mixed with 100 μM biotin alkyne, 1 mM CuSO _4_, 2.5 mM Vc, 1 mM TBTA in sequence and incubated at 27°C, 1000 rpm for 1.5 h. After the click chemistry reaction, 30 μL streptavidin agarose beads were added and incubated at 4°C for 6 h to pulldown the SMO-CP-biotin. The resulting pellet beads were washed with RIPA buffer and boiled at 95°C for 10 min then mixed with membrane solubilization buffer for SDS-PAGE [ 26, 27] .

### Immunoblotting assay

Cells were harvested by centrifugation at 1000 *g* for 5 min, then washed with PBS to remove the redundant medium, followed by homogenization in the RIPA buffer (50 mM Tris-HCl, 150 mM NaCl, 0.1% SDS, 1.5% NP40 and 0.5% deoxycholate, pH 8.0), which contained protease inhibitors (1 mM PMSF, 5 μg/ml pepstatin A, 10 μg/ml leupeptin, 25 μg/ml acetylleucyl-leucyl-norleucine and 10 μM MG132) [28]. Input samples were added in turn to the membrane solubilization buffer (62.5 mM Tris-HCl, 15% SDS, 8 M urea, 10% glycerol, and 100 mM DTT, pH 6.8) and the loading buffer (150 mM Tris-HCl, 12% SDS, 30% glycerol, 6% 2-mercaptoethanol and 0.02% bromophenol blue, pH 6.8). The samples were incubated for 30 min at 37°C. The pellet samples were mixed with 2×loading buffer and boiled at 95°C for 5 min, centrifuged at 12000 *g* for 2 min. Samples were then mixed with an equal volume of the membrane solubilization buffer, and incubated at 37°C for 30 min. Protein samples were resolved by SDS-PAGE and transferred to the PVDF membrane. Membranes were blocked with 2% BSA in TBST, incubated with indicated primary and secondary antibodies. Immunoreactivity was detected by using Pierce ECL substrate (Thermo Scientific).

### Immunofluorescence assay

SMO-KO NIH3T3 cells were washed with PBS, fixed with 4% PFA at room temperature for 30 min, permeabilized with 0.1% Triton X-100 for 5 min, and blocked with 2% BSA. Then, cells were stained with the anti-SMO antibody (1:1000) and anti-ARL13B antibody (1:1000) in 2% BSA and then fluorescent secondary antibodies at room temperature for 1 h. Confocal images were obtained by Leica microscope SP8 (Leica, Heidelberg, Germany). Fluorescence quantification showing the localization of SMO mutants on cilia were performed using Fiji software [29]. For each group, the images of 30 cells were calculated.

### Luciferase assay

For SMO mutants analysis, SMO-KO NIH3T3 cells were plated in 24-well plates and transfected with the plasmids expressing SMO, GliSBS-firefly luciferase and pEGFP-N1 in a ratio of 2 : 2 : 1 with LTX reagent (Thermo Scientific) [30]. Cells were incubated with or without 0.5% serum medium containing N-Shh for 24 h. Each mutant was performed in triplicate [13].

### Sequence analysis

The sequence analysis was performed by using the clustal software to align SMO-CRD sequences [31]. The proteins ID was based on the uniport website.

### Statistical analysis

Data are presented as mean±SD. Statistical analysis was performed in GraphPad Prism 8 software using one-way ANOVA. *P*<0.05 were considered statistically significant.

## Results

### SMO-CRD(27–192) is the minimal segment for cholesterylation

**Figure 1:**
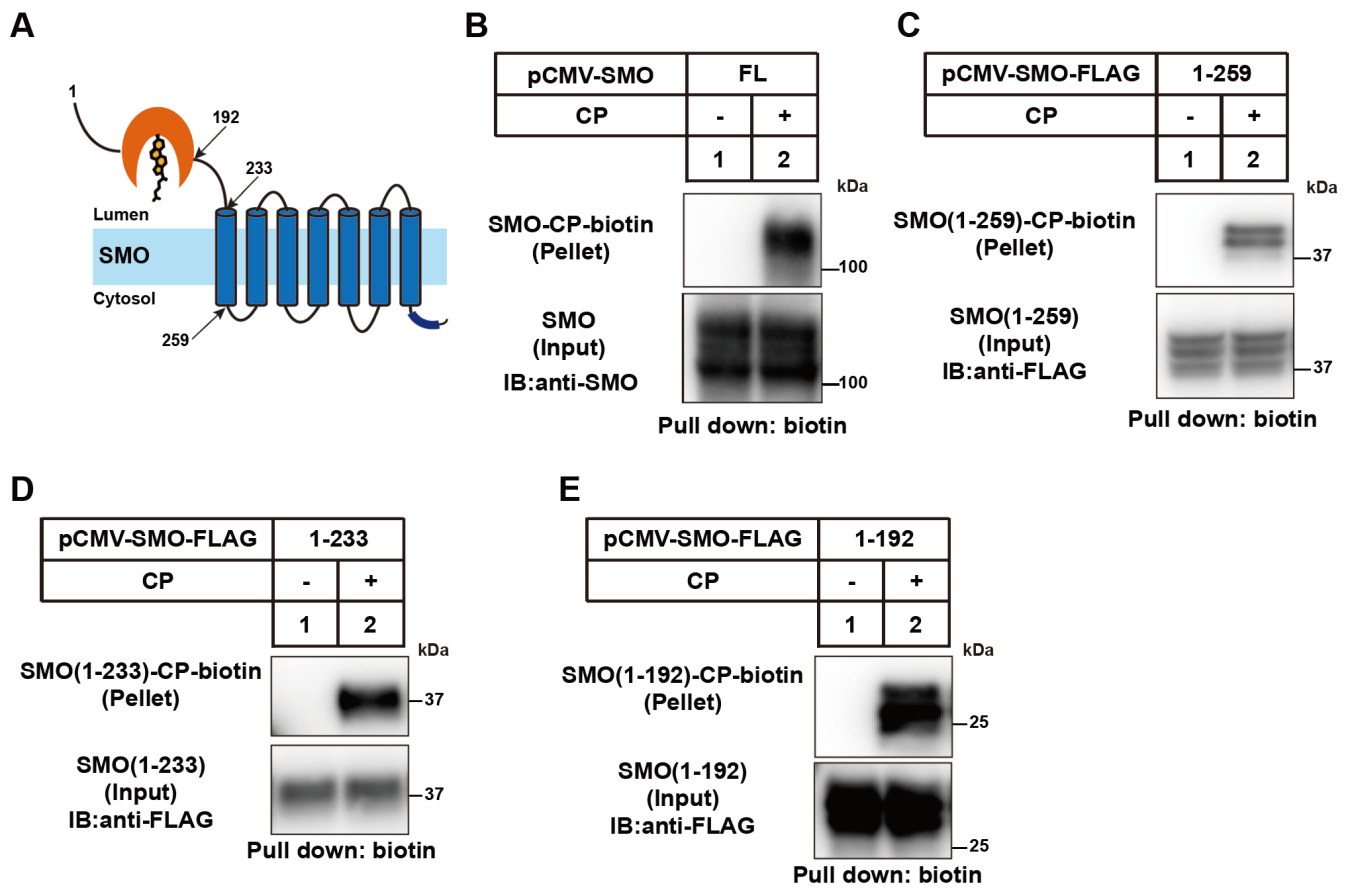
Figure 1 SMO-CRD(27–192) is the minimal segment for SMO cholesterylation (A) The topology of human SMO protein. (B–E) Cholesterylation of the SMO variants. HEK293T cells were transfected with the indicated SMO-expressing plasmids. After incubation with the medium containing 2 μg/mL cholesterol probe (CP) for 16 h, the cells were harvested and subjected to the click assay with biotin-alkyne. The CP-modified SMO was pulled down with neutravidin beads and analyzed by immunoblotting with the anti-SMO antibody.

The topology of human SMO is shown in Figure 1A. To identify the minimal region of SMO that can be modified by cholesterol, we expressed the full-length (FL) and different truncated SMO in HEK293T cells. After incubation in the medium containing cholesterol probe (CP) that is a cholesterol analog with azide group on the side chain [21], the cells were lysed and subjected to click chemistry reaction that allows the linkage with a biotin molecule. The biotinylated SMO protein with CP modification was pulled down using neutravidin agarose and analyzed with anti-SMO antibody. This procedure was described in detail in previous reports [ 9– 21] .

We confirmed that SMO could be modified by CP ( Figure 1B). The truncated SMO proteins including SMO(1 **–**259), SMO(1 **–**233) and SMO(1 **–**192) were all efficiently conjugated by CP ( Figure 1B **–**E). These results are consistent with our previous report showing that SMO cholesterylation is a Ca ^2+^-boosted autoreaction [22].

### The cation-π interaction between Y85 and K133 is essential for cholesterylation of SMO

To understand the molecular details of SMO cholesterylation, we individually mutated the residues in CRD of human SMO that contain functional groups in the side chains. A total of 66 residues, including 15 acidic ones (D95, D97, D137, D165, D172, E71, E100, E101, E135, E140, E158, E160, E176, E181 and E194), 18 basic ones (K105, K133, K186, R28, R42, R49, R50, R66, R74, R113, R117, R138, R144, R151, R159, R161, R168 and R173), 9 aromatic ones (F166, F174, F187, W109, W119, W163, Y75, Y85, and Y130) and 24 polar ones (T37, T55, T88, T90, T145, T150, T170, T179, S32, S33, S43, S47, S51, S62, S81, S89, S96, S98, S110, S143, S189, S190, H63, and H103), were mutated to alanine.

**Figure 2:**
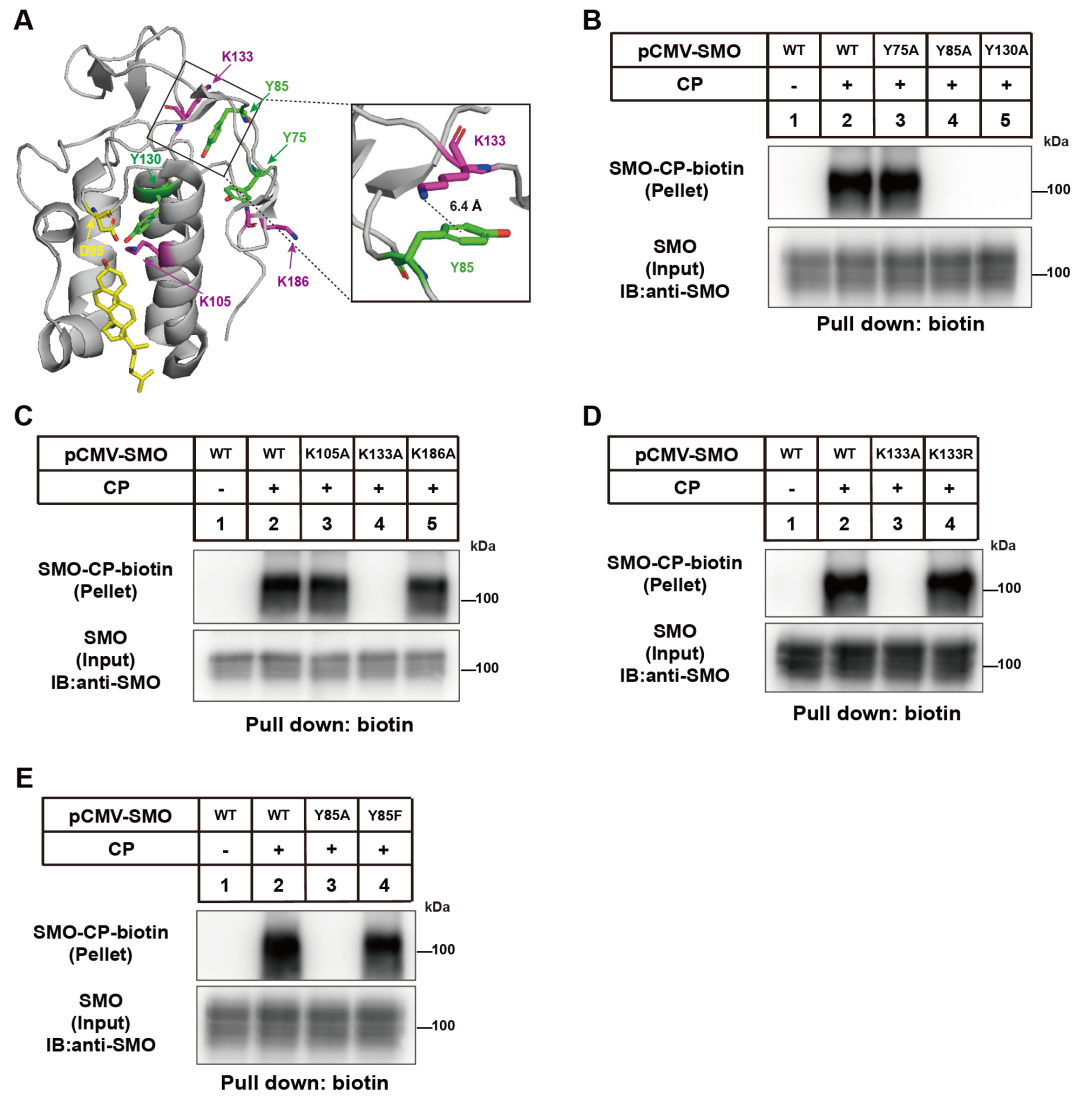
Figure 2 Analysis of the requirement of Y and K in CRD for SMO cholesterylation (A) Illustration of lysine (K, magenta) and tyrosine (Y, green) residues in the SMO-CRD structure based on PDB: 5L7D. The Y85 and K133 residues are enlarged on the right. (B–E) Cholesterylation of SMO variants. The experiments were done as described in Figure1B.

The sequence alignment of SMO-CRD in human, mouse, zebrafish, drosophila and xenopus is shown in Supplementary Figure S1. SMO-CRD contains three tyrosine residues and three lysine residues ( Figure 2A). The cation-π interaction is defined as d1< 7.0 Å [32]. The distance between the ammonium nitrogen of K133 and the centroid of the aromatic ring of Y85 is 6.4 Å, fitting the definition for cation-π interaction. The Y130A mutation abolished SMO cholesterylation ( Figure 2B), which is consistent with our previous findings that the side chains of Y130 and D95 form the D95-Y130 intramolecular ester bond that is required for subsequent ester bonding between cholesterol and D95 [22]. Interestingly, Y85A and K133A mutations also completely abrogated cholesterol modification of SMO ( Figure 2B,C). Since Y85 and K133 are spatially distant from D95 ( Figure 2A), we hypothesize that they may not directly participate in the cholesterylation reaction but interact with each other through cation-π interaction. To test this hypothesis, we replaced K133 with arginine and Y85 with phenylalanine. The SMO(K133R) and SMO(Y85F) could be efficiently conjugated with CP ( Figure 2D,E). Together, these results suggest that Y85 and K133 form a cation-π interaction, which is critical for SMO cholesterylation likely through maintaining the structure of CRD.

### The W109, W119, W163 and F166 residues are critical for glycosylation and cholesterylation of SMO

**Figure 3:**
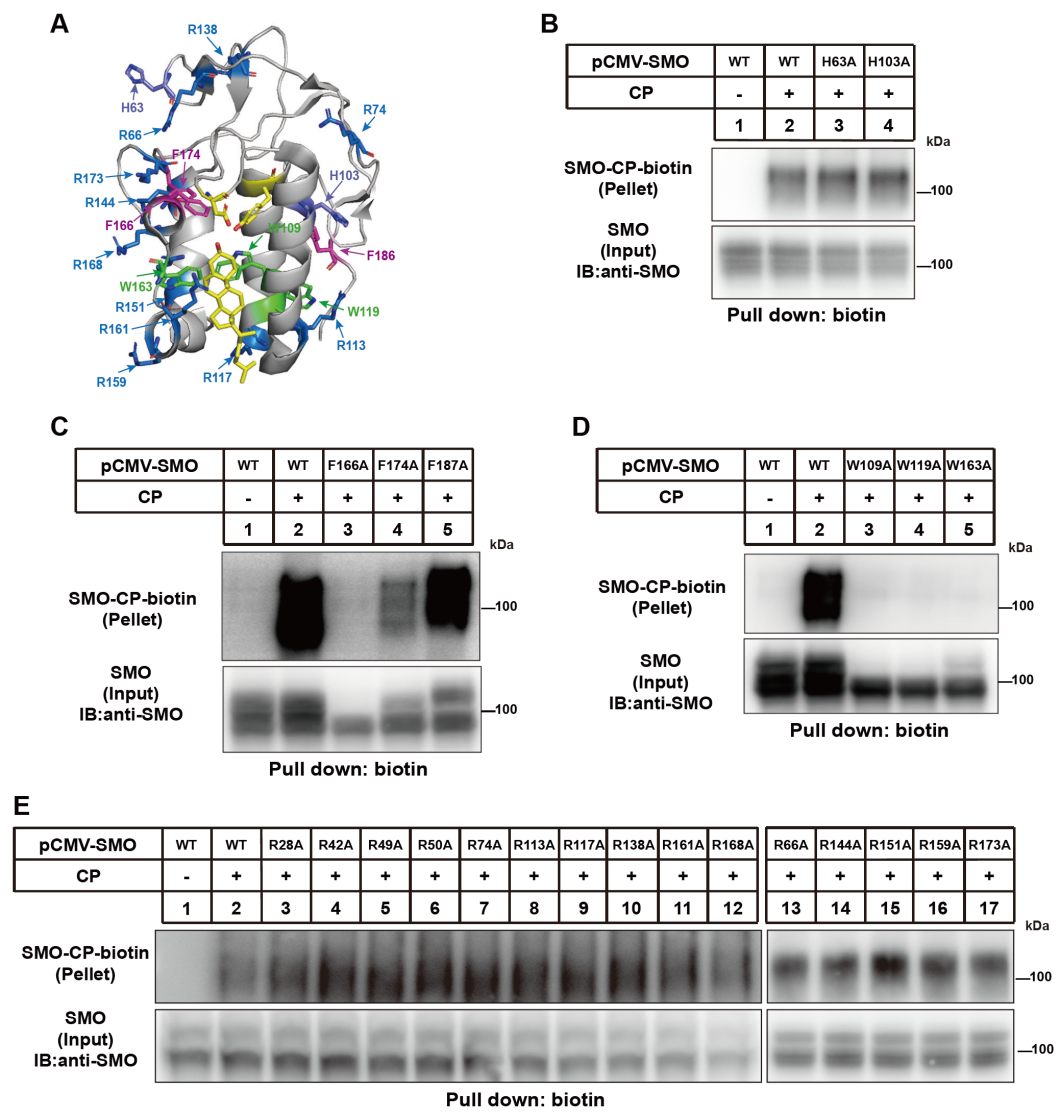
Figure 3 Analysis of the requirement of W, F, H and R residues in CRD for SMO cholesterylation (A) Illustration of tryptophan (W, green), phenylalanine (F, magenta), histidine (H, purple) and arginine (R, blue) residues in the SMO-CRD structure based on PDB: 5L7D. Tryptophan is shown in green. (B–E) Cholesterylation of SMO variants. The experiments were done as described in Figure1B.

Mutation of the two histidine residues and fifteen arginine residues did not affect cholesterylation of SMO ( Figure 3A **–**C). Consistently, their residence is far from the cholesterol-binding groove in the CRD ( Figure 3A). However, replacement of alanine with F166, W109, W119 and W163 nearly eliminated cholesterol modification of SMO ( Figure 3D,E). The F174A mutation partially decreased SMO cholesterylation. The F166A, W109A, W119A and W163A forms of SMO mainly showed one low-molecular-weight band in input fraction, suggesting that they are not efficiently glycosylated and their protein folding might be significantly impaired.

### The E160 residue is critical for SMO cholesterylation

**Figure 4:**
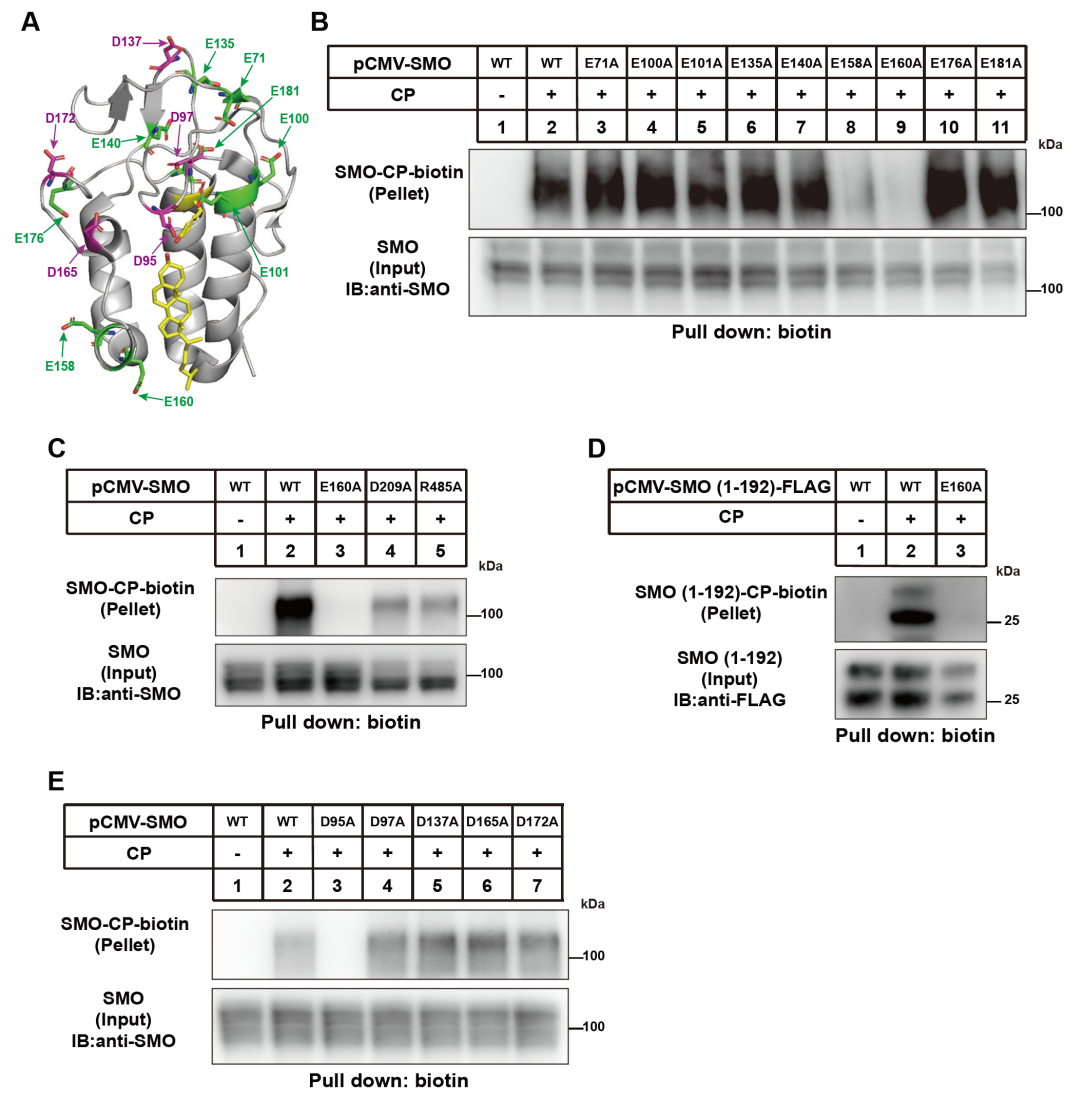
Figure 4 Analysis of the requirement of E and D residues in CRD for SMO cholesterylation (A) Illustration of glutamic acid (E, green) and aspartic acid (D, magenta) in the SMO-CRD structure based on PDB: 5L7D. (B–E) Cholesterylation of SMO variants. The experiments were done as described in Figure1B.

We examined the effect of acidic residues in CRD on SMO cholesterylation. The D95 residue is the cholesterol modification site and D95A mutation prevented CP linkage ( Figure 4A,B). Besides D95A, E158A partially and E160A completely abolished cholesterol modification of SMO ( Figure 4C). In the structure of FL SMO protein [20], R485 is spatially close to E160 and D209. But R485A and D209A mutations only partially decreased SMO modification by cholesterol. Consistent with the findings that SMO(1 **–**192) can be conjugated by cholesterol ( Figure 1E), E160A mutation completely abolished SMO cholesterylation ( Figure 4B **–**E). These results demonstrate that E160 is critical for cholesterol linkage but this effect is not related to R485.

**Figure 5:**
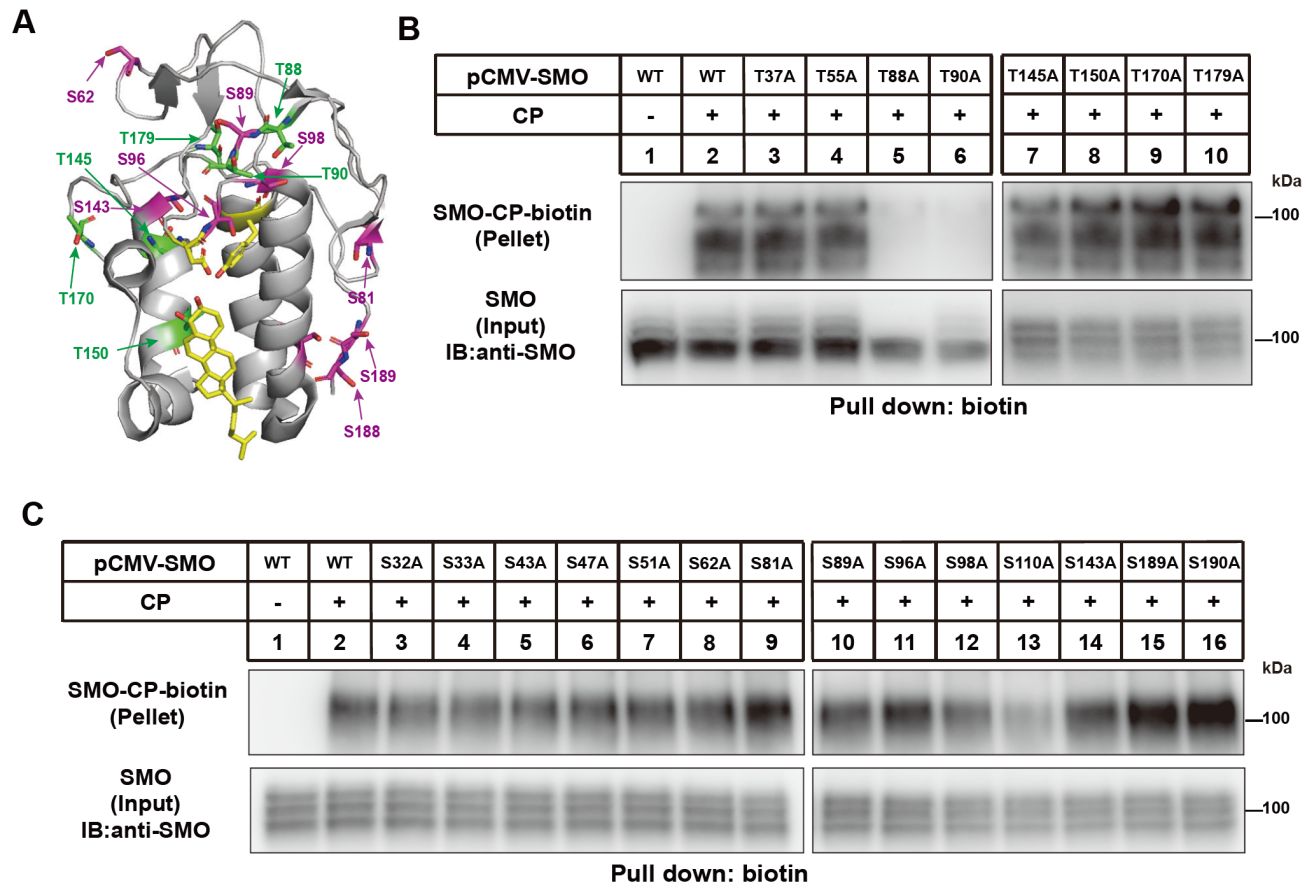
Figure 5 Analysis of the requirement of T and S residues in SMO(1-192) for SMO cholesterylation (A) Illustration of threonine (T, green) and serine (S, magenta) residues in the CRD based on PDB: 5L7D. (B–C) Cholesterylation of SMO variants. The experiments were done as described in Figure1B.

We analyzed whether any threonine or serine residues in CRD are required for SMO cholesterylation. Figure 5 showed that T88 and T90 are essential for SMO modification. The glycosylation of SMO was severely attenuated by T88A but slightly changed by T90A ( Figure 5B). The S110A mutation partially decreased SMO cholesterylation ( Figure 5).

### Correlation between SMO cholesterylation and ciliary localization

**Figure 6:**
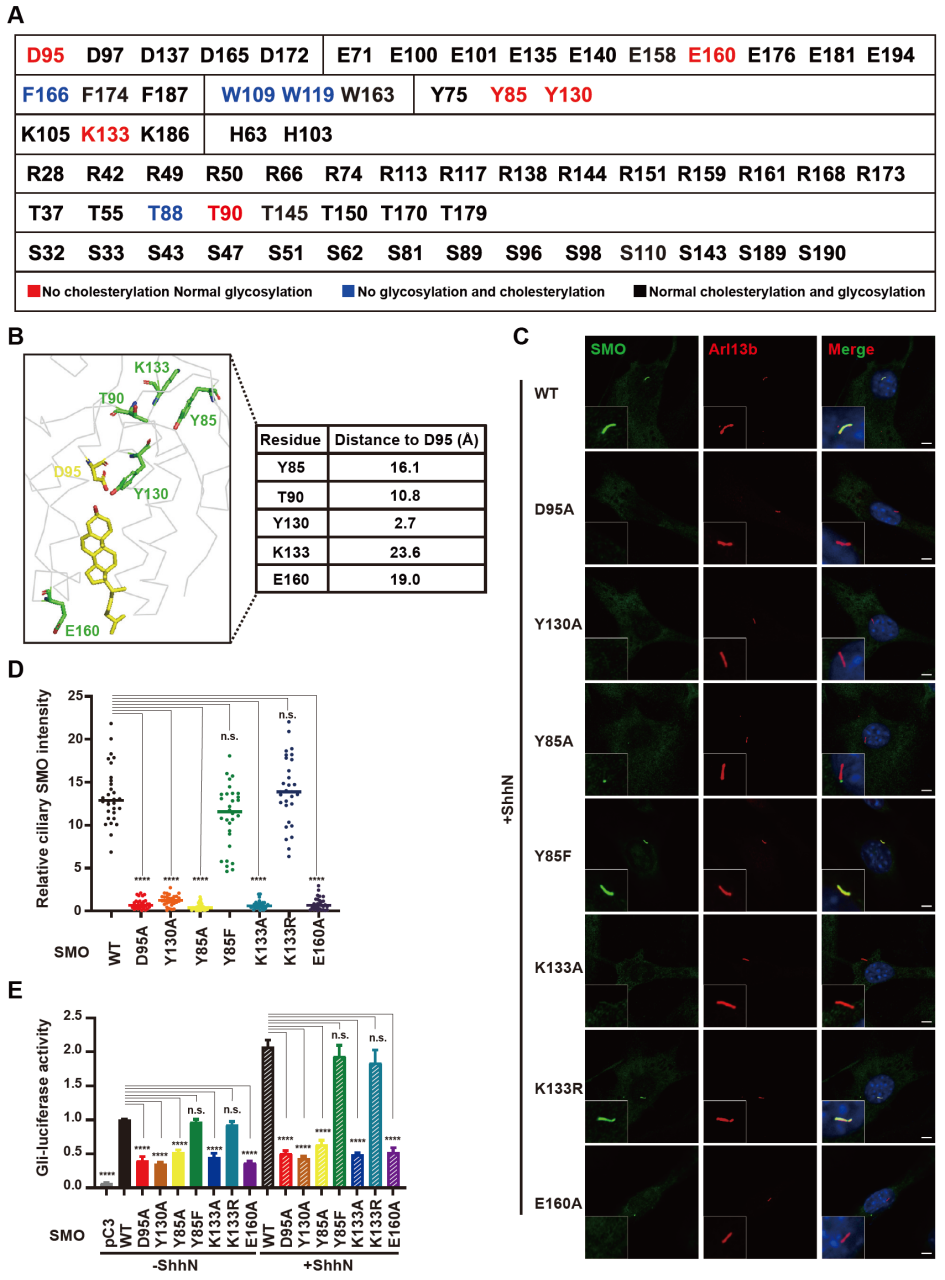
Figure 6 Analysis of the function of SMO variants (A) Summary of the effect of different residues in human SMO(1–192). Mutations of the residues in red abolished the cholesterylation but not glycosylation of SMO. Mutations of the residues in blue abolished both glycosylation and cholesterylation of SMO. Mutations of the residues in black had no obvious effects on the glycosylation or cholesterylation of SMO. (B) The distance of the indicated residues to D95 analyzed by PyMOL software based on PDB: 5L7D. (C) Primary ciliary localization of SMO variants in Smo-deficient NIH3T3 cells treated with ShhN. SMO is shown in green and ARL13B, the cilia marker, is shown in red. Nuclei were counterstained with DAPI. Scale bar, 5 μm. (D) Quantification of SMO intensity on primary cilium (n=30 for each group). Data are presented as mean±SD and analyzed using one-way ANOVA. **** P<0.0001, n.s., not statistically significant. (E) The Gli-Luc reporter assay of SMO variants in Smo-deficient NIH3T3 cells treated with or without ShhN. Data are presented as mean±SD and analyzed using one-way ANOVA. **** P<0.0001, n.s., not statistically significant.

Based on how alanine substitution affects cholesterylation and protein glycosylation, we divided the 66 residues examined earlier into three categories. Class 1 included the residues whose mutation abolished cholesterylation but not glycosylation appearance of SMO ( Figure 6A, in red). The residues in class 2 were essential for both glycosylation and cholesterylation ( Figure 6A, in blue), suggesting their critical roles in protein folding. The class 3 residues showed normal cholesterylation and glycosylation when mutated to alanine ( Figure 6A, in black). We focused on the residues in class 1 because their mutations seem not to affect protein folding. Figure 6B showed their location relative to D95, where cholesterylation occurs.

To analyze the function of SMO variants, we transiently expressed them in *Smo*-deficient NIH3T3 cells. ShhN induced the ciliary localization of SMO(Y85F) and SMO(K133R), both of which could be highly cholesterylated, as that of WT SMO. The Y130A, Y85A, K133A, and E160A mutants, like the D95A mutation, were barely translocated to primary cilium ( Figure 6C,D). Gli luciferase reporter assay also confirmed that the D95A, Y130A, Y85A, K133A, E160A mutants substantially lost the activity of signal transduction under both basal and N-Shh-stimulating conditions ( Figure 6E) , suggesting that cholesterol modification is a prerequisite for SMO translocation to primary cilium and critical for Hh signal transduction.

## Discussion

The human SMO protein is covalently linked to cholesterol on the D95 residue and this cholesterylation is critical for the SMO activation by Hh [ 21, 22] . Our previous work demonstrated that SMO cholesterylation is a Ca ^2+^-boosted autoreaction. Cholesterol occupies the groove of SMO-CRD with 3-beta hydroxyl group residing adjacent to D95. The side chains of D95 and Y130 form an ester bond that serves as a high energy intermediate. Then the 3-beta hydroxyl group of cholesterol forms an ester bond with the side chain of D95.

In this study, we systematically analyzed the residues of SMO(27-192) that harbor functional groups. We analyzed the effects of 66 residues on SMO cholesterylation. Besides D95 and Y130, eight additional residues (E160, Y85, K133, T90, F166, W109, W119 and T88) were identified to be critical for cholesterol modification. The F166, W109, W119 and T88 residues also affect glycosylation of SMO. Therefore, it is possible that their impairment of cholesterylation is a secondary consequence of protein misfolding. The distance of other residues to D95 is more than 10.8 Å ( Figure 6B), suggesting they may not directly participate in ester bond formation.

Although we do not know how mutations of E160 and T90 abolish SMO cholesterylation, our results suggest Y85 and K133 form a cation-π pair that is required for cholesterol conjugation to SMO and ciliary localization of SMO. The cation-π interaction is a major force determining molecule interaction and protein conformation. In the SMO protein, a short α-helical structure proceeding the C295 residue in SMO is stabilized by a cation-π interaction [22]. The cation-π interaction between TM6 and TM7 is essential for locking SMO in an inactive configuration and opens in the active state [24]. In this study, we identify a cation-π interaction inside the CRD of SMO. In contrast to the cation-π interaction between TM6 and TM7, this new cation-π pair between Y85 and K133 is required for SMO cholesterylation and activation. Importantly, it is worth further investigation whether these residues critical for SMO cholesterylation are subjected to other types of modification or regulation so that the Hh signaling can be modulated.

## Supplementary Data

Supplementary Data is available at *Acta Biochimica et Biphysica Sinica* online.

## References

[1] Bale AE (2002). Hedgehog signaling and human disease. Annu Rev Genom Hum Genet.

[2] Varjosalo M, Taipale J (2008). Hedgehog: functions and mechanisms. Genes Dev.

[3] Hu A, Song BL (2019). The interplay of Patched, Smoothened and cholesterol in Hedgehog signaling. Curr Opin Cell Biol.

[4] Teglund S, Toftgård R (2010). Hedgehog beyond medulloblastoma and basal cell carcinoma. Biochim Biophys Acta (BBA) - Rev Cancer.

[5] Ingham PW, McMahon AP (2001). Hedgehog signaling in animal development: paradigms and principles. Genes Dev.

[6] Pak E, Segal RA (2016). Hedgehog signal transduction: key players, oncogenic drivers, and cancer therapy. Dev Cell.

[7] Qiu ZP, Hu A, Song BL (2021). The 3-beta-hydroxysteroid-Delta(8), Delta(7)-isomerase EBP inhibits cholesterylation of Smoothened. Biochim Biophys Acta (BBA) - Mol Cell Biol Lipids.

[8] Rohatgi R, Milenkovic L, Scott MP (2007). Patched1 regulates hedgehog signaling at the primary cilium. Science.

[9] Hu A, Zhou M, Song BL. Analysis of protein cholesterylation by biorthogonal labeling. Methods in molecular biology (Clifton, N.J.) 2022, 2374: 27–36.

[10] Corbit KC, Aanstad P, Singla V, Norman AR, Stainier DYR, Reiter JF (2005). Vertebrate Smoothened functions at the primary cilium. Nature.

[11] Huangfu D, Liu A, Rakeman AS, Murcia NS, Niswander L, Anderson KV (2003). Hedgehog signalling in the mouse requires intraflagellar transport proteins. Nature.

[12] Xie J, Murone M, Luoh SM, Ryan A, Gu Q, Zhang C, Bonifas JM (1998). Activating Smoothened mutations in sporadic basal-cell carcinoma. Nature.

[13] Huang P, Nedelcu D, Watanabe M, Jao C, Kim Y, Liu J, Salic A (2016). Cellular cholesterol directly activates smoothened in hedgehog signaling. Cell.

[14] Deshpande I, Liang J, Hedeen D, Roberts KJ, Zhang Y, Ha B, Latorraca NR (2019). Smoothened stimulation by membrane sterols drives Hedgehog pathway activity. Nature.

[15] Qi X, Liu H, Thompson B, McDonald J, Zhang C, Li X (2019). Cryo-EM structure of oxysterol-bound human Smoothened coupled to a heterotrimeric Gi. Nature.

[16] Huang P, Zheng S, Wierbowski BM, Kim Y, Nedelcu D, Aravena L, Liu J (2018). Structural basis of smoothened activation in hedgehog signaling. Cell.

[17] Luchetti G, Sircar R, Kong JH, Nachtergaele S, Sagner A, Byrne EF, Covey DF (2016). Cholesterol activates the G-protein coupled receptor Smoothened to promote Hedgehog signaling. eLife.

[18] Byrne EFX, Sircar R, Miller PS, Hedger G, Luchetti G, Nachtergaele S, Tully MD (2016). Structural basis of Smoothened regulation by its extracellular domains. Nature.

[19] Rana R, Carroll CE, Lee HJ, Bao J, Marada S, Grace CRR, Guibao CD (2013). Structural insights into the role of the Smoothened cysteine-rich domain in Hedgehog signalling. Nat Commun.

[20] Qi X, Friedberg L, De Bose-Boyd R, Long T, Li X (2020). Sterols in an intramolecular channel of Smoothened mediate Hedgehog signaling. Nat Chem Biol.

[21] Xiao X, Tang JJ, Peng C, Wang Y, Fu L, Qiu ZP, Xiong Y (2017). Cholesterol modification of smoothened is required for hedgehog signaling. Mol Cell.

[22] Hu A, Zhang JZ, Wang J, Li CC, Yuan M, Deng G, Lin ZC (2022). Cholesterylation of Smoothened is a calcium-accelerated autoreaction involving an intramolecular ester intermediate. Cell Res.

[23] Wang LJ, Wang J, Li N, Ge L (2011). Molecular characterization of the NPC1L1 variants identified from cholesterol low absorbers. J Biol Chem.

[24] Bok J W, Keller N P. Fast and easy method for construction of plasmid vectors using modified quick-change mutagenesis. Methods in Molecular Biology (Clifton, N.J.) 2012, 944: 163–174.

[25] Tang JJ, Li JG, Qi W, Qiu WW, Li PS, Li BL, Song BL (2011). Inhibition of SREBP by a small molecule, betulin, improves hyperlipidemia and insulin resistance and reduces atherosclerotic plaques. Cell Metab.

[26] Roux KJ, Kim DI, Raida M, Burke B (2012). A promiscuous biotin ligase fusion protein identifies proximal and interacting proteins in mammalian cells. J Cell Biol.

[27] Wei J, Fu ZY, Li PS, Miao HH, Li BL, Ma YT, Song BL (2014). The clathrin adaptor proteins ARH, Dab2, and numb play distinct roles in Niemann-Pick C1-Like 1 versus low density lipoprotein receptor-mediated cholesterol uptake. J Biol Chem.

[28] Lu XY, Shi XJ, Hu A, Wang JQ, Ding Y, Jiang W, Sun M (2020). Feeding induces cholesterol biosynthesis via the mTORC1–USP20–HMGCR axis. Nature.

[29] Smith S, Septer AN (2021). Quantification of interbacterial competition using single-cell fluorescence imaging. JoVE.

[30] Fu L, Wu H, Cheng SY, Gao D, Zhang L, Zhao Y (2016). Set7 mediated Gli3 methylation plays a positive role in the activation of Sonic Hedgehog pathway in mammals. eLife.

[31] Marada S, Navarro G, Truong A, Stewart DP, Arensdorf AM, Nachtergaele S, Angelats E (2015). Functional divergence in the role of N-linked glycosylation in smoothened signaling. PLoS Genet.

[32] Xu Z, Zhang Q, Shi J, Zhu W (2019). Underestimated noncovalent interactions in protein data bank. J Chem Inf Model.

